# Dysfunction of Immune Systems and Host Genetic Factors in Hepatitis C Virus Infection with Persistent Normal ALT

**DOI:** 10.1155/2011/713216

**Published:** 2011-06-14

**Authors:** Yasuteru Kondo, Yoshiyuki Ueno, Tooru Shimosegawa

**Affiliations:** Division of Gastroenterology, Tohoku University Hospital, 1-1 Seiryo-Machi, Aoba-ku, Sendai City, Miyagi 980-8574, Japan

## Abstract

Patients with chronic hepatitis C (CHC) virus infection who have persistently normal alanine aminotransferase levels (PNALT) have mild inflammation and fibrosis in comparison to those with elevated ALT levels. The cellular immune responses to HCV are mainly responsible for viral clearance and the disease pathogenesis during infection. However, since the innate and adaptive immune systems are suppressed by various kinds of mechanisms in CHC patients, the immunopathogenesis of CHC patients with PNALT is still unclear. In this review, we summarize the representative reports about the immune suppression in CHC to better understand the immunopathogenesis of PNALT. Then, we summarize and speculate on the immunological aspects of PNALT including innate and adaptive immune systems and genetic polymorphisms of HLA and cytokines.

## 1. Introduction

Hepatitis C virus (HCV) is noncytopathic virus that causes chronic hepatitis and hepatocellular carcinoma (HCC) [1]. Approximately 70–80% of those acutely infected will become persistently infected with HCV [1]. Around 30% of CHC patients exhibit PNALT and show milder disease activity and slower progression to hepatic cirrhosis [2–6]. However, it is reported that about 40% of these progress to the active stage of inflammation, but the incidence of HCC in the PNALT was lower than that in those with elevated ALT levels [7]. Cellular and humoral immune responses to HCV play an important role in the pathogenesis of active and nonactive chronic hepatitis [8].

Numerous studies have indicated that failure of the cellular immune response, including type 1 helper T cells (Th1) hypo-responsiveness, cytotoxic T lymphocyte (CTL) exhaustion, excessive function of CD4+  CD25+  FOXP3+ regulatory T cells, and failure of lymphoid cells via direct binding and/or infection in B cells, T cells, NK cells, and DCs occurs in CHC patients [9–21]. Since the liver damage in CHC is mainly induced by Th1 and/or CTL related responses [22–24], these responses might be strongly suppressed in PNALT.

 Given the involvement of immune responses, genetic factors including polymorphism of HLA and cyotokine-related genes could also contribute to the activity of inflammation in CHC patients [25–37]. Many groups, including us, have reported on the relationship between certain HLA and ALT levels in CHC patients [25, 27–29]. Moreover, some groups indicated the polymorphism of certain cytokines-related genes contributed to the level of inflammation [30–37]. In a genomewide association study (GWAS), IL28B polymorphism was shown to influence the outcome of Peg-IFN and RBV therapy [38]. However, although the relationship between IL28B polymorphism and PNALT is still unclear, a possible relationship between IL28B polymorphism and the activity of inflammation has been reported [39, 40].

In this review, we focused on the possible immunopathological aspects of PNALT and summarize various studies about the mechanism and genetic host factors involved in the immune suppression in CHC patients. 

## 2. Mechanism of Immune Suppression in CHC Patients

### 2.1. Innate Immune System in Hepatocytes

#### 2.1.1. Immune Suppression in CH-C

**Figure 1:**
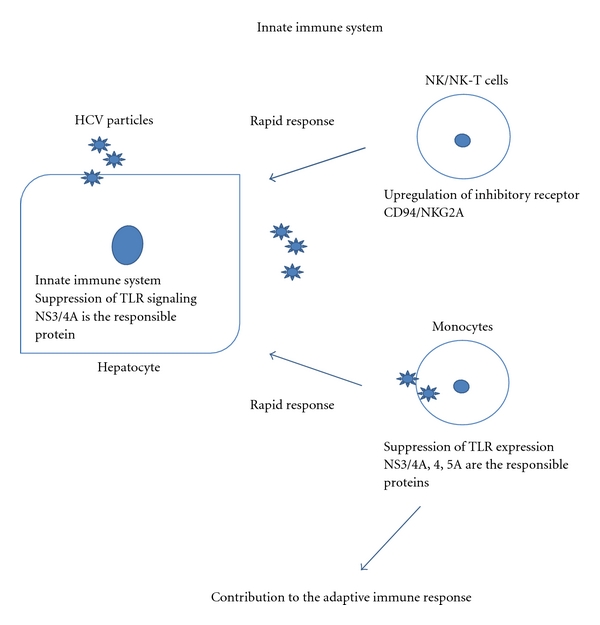
The schema of innate immune suppression in CHC is shown. The representative suppressive mechanisms are shown in this figure.

Innate immune systems are important for the initial step of viral infection [41, 42] (Figure 1). Toll-like receptors (TLRs) are a family of innate immune-recognition receptors that recognize molecular patterns associated with microbial pathogens including single and double-strand RNA. HCV interferes with the innate immune response and the induction of IFN-beta via the HCV NS3/4A protease activity, which inhibits the phosphorylation of IRF-3, a key transcriptional regulator of the IFN response [43, 44]. HCV NS3 protein interacts directly with TBK1, and this binding results in the inhibition of the association between TBK1 and IRF-3, which leads to the inhibition of IRF-3 activation [45].

#### 2.1.2. Immune Suppression in PNALT

However, since little is known about the direct relationship between the suppression of TLR signaling and PNALT, the level of TLR signaling suppression might contribute to the immunopathogenesis of PNALT.

### 2.2. Monocyte, NK, and NK-T Cells

#### 2.2.1. Immune Suppression in CH-C

In addition to the intracellular immune reaction of hepatocytes, monocytes, NK, and NK-T cells are responsible for the rapid reaction in HCV infection (Figure 1). Many groups have described that NK and NK-T cells were suppressed in CHC patients [46–53]. However, whether NK and NK-T cells would be suppressed in CHC patients is still controversial [48]. The various backgrounds of CHC patients have resulted in controversial findings [49]. The function of NK cells is regulated by a balance of inhibitory and activating signals, which are mediated by the differential expression of receptors. Takehara and Hayashi recently reported that the expression of the inhibitory receptor CD94/NKG2A is upregulated on NK cells in CHC patients [53]. Another group reported that exposure to HCVcc modulates the pattern of cytokines produced by NK cells, leading to reduced antiviral activity [47].

#### 2.2.2. Immune Suppression in PNALT

Previously, it was reported that HCV-core and NS3 protein triggered the inflammatory pathway via TLR2, which might affect viral recognition and activation of the immune system [54]. It was also reported that peripheral blood monocyte expression of TLR2 but not of TLR4 correlated significantly with the serum ALT levels [55]. Concerning the mechanisms of monocyte-suppression, it was clearly demonstrated that macrophage cell lines expressing NS3, NS3/4A, NS4B, or NS5A inhibited the activation of the TLR2, TLR4, TLR7, and TLR9 signaling pathways. Among the HCV individual proteins, NS5A bound to MyD88, a major adaptor molecule in TLR, inhibited the recruitment of interleukin-1 receptor-associated kinase 1 to MyD88 and impaired the cytokine production in response to TLR ligands [56]. These results indicated that the activation of monocytes was probably suppressed in low ALT and PNALT CHC patients.

### 2.3. Adaptive Immune Systems: T Cells, B Cells, and Dendritic Cells

#### 2.3.1. Immune-Suppression in CH-C

After the innate immune period, the adaptive immune system including CD4+ T cells, CD8+ cyototoxic T cells, B cells, and dendritic cells should be involved in a more effective immune response to HCV.

**Figure 2:**
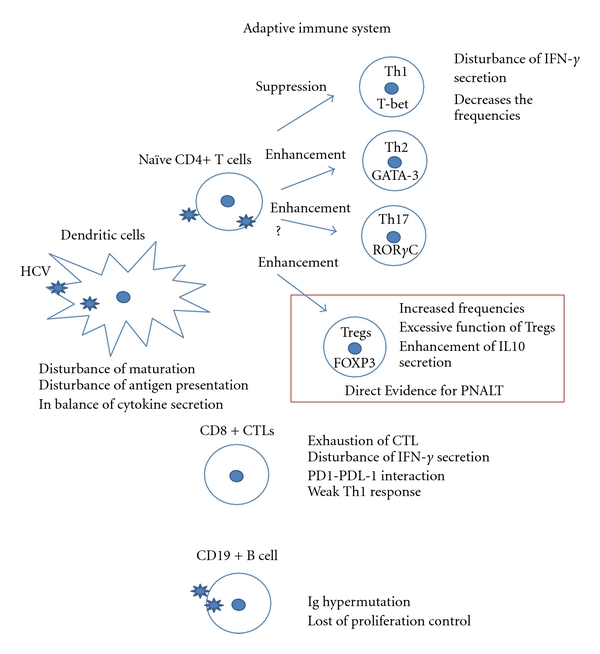
The schema of adaptive immune suppression in CHC is shown. The representative suppressive mechanisms are shown in this figure.

The HCV antigen-driven proliferation of CD4+ T cells is weak in patients who develop persistent HCV infection [57, 58]. It has been demonstrated that depletion of CD4+ T cells results in a weak CD8+ T cell response, which partly controls viremia, followed by viral persistence in chimpanzee infection studies [59]. In addition, an appropriate Th1 response is essential to eradicate HCV [60]. It has been reported that an increased Th2 cytokine response may reduce the inflammatory and biochemical activity [61]. Moreover, various studies have indicated that failure of the adaptive immune response, including Th1 hypo-responsiveness, CD8+ CTL exhaustion, excessive function of CD4+ CD25+ FOXP3+ regulatory T cells, and failure of lymphoid cell via direct binding and/or infection in B cells, T cells, and DCs occurs in CHC patients [9–21]. Previously, we reported the direct suppressive effect of HCV on T cell- and B cell-immunity in CHC by using a lymphotropic HCV strain [14–17]. However, since the contribution of lymphotropic HCV to the pathogenesis of PNALT is still not clear, the biological significance of lymphotropic HCV needs to be analyzed in future studies. Studies about the relationship between the infectivity of lymphotropic HCV and PNALT are ongoing in our laboratory. Tregs constitutively express CD25 (the IL2 receptor alpha-chain) in the physiological state [62]. In human, this Tregs population, defined as CD4+ CD25+ FOXP3+ cells, constitutes 5% to 10% of peripheral CD4+ T cells and has a broad repertoire that recognizes various types of self and nonself antigen. Antigens induced by HCV might induce Tregs to escape from immunological pressure as reported in persistent infection of EB virus, hepatitis B virus, and HIV [63–67] (Figure 2).

#### 2.3.2. Immune Suppression in PNALT

More recently, Itose et al. reported that the frequencies of naturally occurring-Treg in CHC patients were significantly higher than those in healthy individuals. The FOXP3 and CTLA4 transcripts were higher in PNALT than those in CHC patients [21]. Another group also described that their suppressor ability is stronger in patients with PNALT than that in those with active CHC hepatitis [68]. These two studies could be direct evidence about the relationship between PNALT and the function of Tregs. Moreover, a unique subset of lymphocytes might contribute to the immune suppression in PNALT [69] (Figure 2). 

## 3. HLA and Cytokine-Related Gene Polymorphism and ALT Level

### 3.1. HLA Polymorphism

CD8+ CTLs are able to recognize viral antigens synthesized within infected cells in the form of short peptides associated with HLA class I molecules [70]. On the other hand, CD4+ Th cells are able to recognize antigens associated with HLA class II molecules [70, 71]. Individuals that are heterozygous at HLA class I  loci are able to present a greater variety of antigenic peptides to CTL resulting in a broader immune response [72]. HLA class I heterozygosity was found at a higher rate in patients with slow progression to AIDS in HIV-1 infection [73]. However, HLA class I heterozygosity did not affect the inflammatory levels of CHC patients in our previous study [27]. The frequency of HLA-A2 tended to be higher in patients with PNALT than in those with elevated ALT level [27]. A specific HLA class II allele has been reported to influence the disease severity [25] or viral clearance in chronic hepatitis C [26]. Yoshizawa et al. reported that the frequencies of DRB1*12 (*1201 and *1202), DQB1*0301, and DRB3*03 alleles were higher in patients with asymptomatic HCV carriers than those in liver cirrhosis patients [28]. Large-scale studies might be able to verify the ethnic, gender, age, and other genetic factors and determine the influence of the HLA allele on PNALT. 

### 3.2. Cytokine-Related Gene Polymorphism

Numerous studies indicated that various kinds of cytokine-related gene polymorphism were involved in the immunopathogenesis of CHC. IL10 is a suppressive cytokine that could contribute to the persistence of HCV infection and low inflammation level in CHC. Mangia et al. reported that the IL-10 ATA haplotype was more frequent in patients with spontaneous HCV RNA clearance (36.0%) than that in patients with persistent infection (23%) [74]. On the other hand, another group reported that no effect of IL-1beta and IL-10 gene polymorphism on the degree of hepatocellular injury was apparent based on the ALT levels [37]. However, a gender effect is clearly observed in women carrying the GG high IL-10 producer genotype. The higher levels of IL-10 present in such individuals are associated with a higher risk of inefficient clearance of the HCV and the development of a chronic HCV infection together with a lower risk of progression to cirrhosis in female patients [75]. These reports indicated that IL-10-related gene polymorphism might affect the level of inflammation in certain conditions. Another important gene is CCR5Delta32. CCR5Delta32, a 32-base pair deletion of the CC chemokine receptor (CCR) 5 gene, is associated with slower human immunodeficiency virus disease progression in heterozygotes and protection against infection in homozygotes. Goulding et al. reported that heterozygosity for CCR5delta32 was significantly associated with spontaneous hepatitis C viral clearance and with significantly lower hepatic inflammatory scores [33]. In addition to these gene polymorphisms, polymorphism of TGF-beta, TNF-alfa, IL2, IFN-g, and OAS-1 genes might contribute to the level of inflammation [30–32, 35, 74]. More recently, GWAS revealed promising results. A genomewide association of IL28B with the response to pegylated interferon and ribavirin was reported [38]. Then, another group reported that different cytokine profiles induced by the IL28 polymorphism resulted in different Interferon stimulated genes and IL28 expression during chronic HCV infection [76]. They reported that the expression of IL28, MxA, PKR, OAS1, and ISG15 in hepatic cells was significantly lower in patients with the response-favorable (rs8099917) T/T genotype compared to those with T/G or G/G genotypes [76]. A future study might be able to determine the relationship between IL28 polymorphism and PNALT.

## 4. Concluding Remarks

In CHC patients, there are many kinds of immune-suppressive mechanisms. However, although the immunopathogenesis of PNALT has not been clarified yet, the complexities of immune reactions likely contribute to the difficulties of determining the detailed mechanisms of PNALT. Recently, the technologies of GWAS, immunoassay with increased numbers of multicolor flow cytometry analysis, and chimera mice with human hepatocytes and lymphocytes have been developed. These technologies, together with previous data, might be able to clarify the immunopathogenesis of PNALT in CHC.

## References

[1] Alter MJ, Kruszon-Moran D, Nainan OV (1999). The prevalence of hepatitis C virus infection in the United States, 1988 through 1994. *The New England Journal of Medicine*.

[2] Marcellin P, Levy S, Erlinger S (1997). Therapy of hepatitis C: patients with normal aminotransferase levels. *Hepatology*.

[3] Tassopoulos NC (1999). Treatment of patients with chronic hepatitis C and normal ALT levels. *Journal of Hepatology*.

[4] Zapata R (2010). Clinical approach to the patient with chronic hepatitis C infection and normal aminotransferases. *Annals of Hepatology*.

[5] Puoti C, Bellis L, Galossi A (2008). Antiviral treatment of HCV carriers with persistently normal ALT levels. *Mini-Reviews in Medicinal Chemistry*.

[6] Puoti C, Bellis L, Castellacci R (2005). Viral kinetics during antiviral therapy in patients with chronic hepatitis C and persistently normal ALT levels. *Hepatology*.

[7] Persico M, Persico E, Suozzo R (2000). Natural history of hepatitis C virus carriers with persistently normal aminotransferase levels. *Gastroenterology*.

[8] Chang KM, Rehermann B, Chisari FV (1997). Immunopathology of hepatitis C. *Springer Seminars in Immunopathology*.

[9] Accapezzato D, Francavilla V, Paroli M (2004). Hepatic expansion of a virus-specific regulatory CD8^+^ T cell population in chronic hepatitis C virus infection. *Journal of Clinical Investigation*.

[10] Manigold T, Racanelli V (2007). T-cell regulation by CD4 regulatory T cells during hepatitis B and C virus infections: facts and controversies. *Lancet Infectious Diseases*.

[11] Blackburn SD, Wherry EJ (2007). IL-10, T cell exhaustion and viral persistence. *Trends in Microbiology*.

[12] Blackburn SD, Crawford A, Shin H, Polley A, Freeman GJ, Wherry EJ (2010). Tissue-specific differences in PD-1 and PD-L1 expression during chronic viral infection: implications for CD8 T-cell exhaustion. *Journal of Virology*.

[13] Yao ZQ, Prayther D, Trabue C, Dong ZP, Moorman J (2008). Differential regulation of SOCS-1 signalling in B and T lymphocytes by hepatitis C virus core protein. *Immunology*.

[14] Kondo Y, Sung VMH, Machida K, Liu M, Lai MMC (2007). Hepatitis C virus infects T cells and affects interferon-*γ* signaling in T cell lines. *Virology*.

[15] Kondo Y, Machida K, Liu HM (2009). Hepatitis C virus infection of T cells inhibits proliferation and enhances Fas-mediated apoptosis by down-regulating the expression of CD44 splicing variant 6. *Journal of Infectious Diseases*.

[16] Kondo Y, Ueno Y, Kakazu E (2011). Lymphotropic HCV strain can infect human primary naïve CD4^+^ cells and affect their proliferation and IFN-*γ* secretion activity. *Journal of Gastroenterology*.

[17] Machida K, Kondo Y, Huang JY (2008). Hepatitis C virus (HCV)-induced immunoglobulin hypermutation reduces the affinity and neutralizing activities of antibodies against HCV envelope protein. *Journal of Virology*.

[18] Jinushi M, Takehara T, Kanto T (2003). Critical role of MHC class I-related chain A and B expression on IFN-*α*-stimulated dendritic cells in NK cell activation: impairment in chronic hepatitis C virus infection. *Journal of Immunology*.

[19] Kanto T, Hayashi N, Takehara T (1999). Impaired allostimulatory capacity of peripheral blood dendritic cells recovered from hepatitis C virus-infected individuals. *Journal of Immunology*.

[20] Kanto T, Inoue M, Miyatake H (2004). Reduced numbers and impaired ability of myeloid and plasmacytoid dendritic cells to polarize T helper cells in chronic hepatitis C virus infection. *Journal of Infectious Diseases*.

[21] Itose I, Kanto T, Kakita N (2009). Enhanced ability of regulatory T cells in chronic hepatitis C patients with persistently normal alanine aminotransferase levels than those with active hepatitis. *Journal of Viral Hepatitis*.

[22] Nelson DR, Marousis CG, Davis GL (1997). The role of Hepatitis C virus-specific cytotoxic T lymphocytes in chronic hepatitis C. *Journal of Immunology*.

[23] Nelson DR, Marousis CG, Ohno T, Davis GL, Lau JYN (1998). Intrahepatic hepatitis C virus-specific cytotoxic T lymphocyte activity and response to interferon alfa therapy in chronic hepatitis C. *Hepatology*.

[24] Schirren CA, Jung MC, Gerlach JT (2000). Liver-derived hepatitis C virus (HCV)-specific CD4(+) T cells recognize multiple HCV epitopes and produce interferon gamma. *Hepatology*.

[25] Kuzushita N, Hayashi N, Moribe T (1998). Influence of HLA haplotypes on the clinical courses of individuals infected with hepatitis C virus. *Hepatology*.

[26] Renou C, Halfon P, Pol S (2002). Histological features and HLA class II alleles in hepatitis C virus chronically infected patients with persistently normal alanine aminotransferase levels. *Gut*.

[27] Kondo Y, Kobayashi K, Kobayashi T (2003). Distribution of the HLA class I allele in chronic hepatitis C and its association with serum ALT level in chronic hepatitis C. *Tohoku Journal of Experimental Medicine*.

[28] Yoshizawa K, Ota M, Saito S (2003). Long-term follow-up of hepatitis C virus infection: HLA class II loci influences the natural history of the disease. *Tissue Antigens*.

[29] Yu RB, Hong X, Ding WL (2008). The association between the genetic polymorphism of HLA-DQA1, DQB1, and DRB1 and serum alanine aminotransferase levels in chronic hepatitis C in the Chinese population. *Journal of Gastroenterology and Hepatology*.

[30] Li CZ, Kato N, Chang JH (2009). Polymorphism of OAS-1 determines liver fibrosis progression in hepatitis C by reduced ability to inhibit viral replication. *Liver International*.

[31] Gao QJ, Liu DW, Zhang SY (2009). Polymorphisms of some cytokines and chronic hepatitis B and C virus infection. *World Journal of Gastroenterology*.

[32] Dai CY, Chuang WL, Lee LP (2006). Associations of tumour necrosis factor alpha promoter polymorphisms at position -308 and -238 with clinical characteristics of chronic hepatitis C. *Journal of Viral Hepatitis*.

[33] Goulding CA, McManus R, Murphy A (2005). The CCR5-Δ32 mutation: impact on disease outcome in individuals with hepatitis C infection from a single source. *Gut*.

[34] Mochida S, Hashimoto M, Matsui A (2004). Genetic polymorphims in promoter region of osteopontin gene may be a marker reflecting hepatitis activity in chronic hepatitis C patients. *Biochemical and Biophysical Research Communications*.

[35] Ben-Ari Z, Pappo O, Druzd T (2004). Role of cytokine gene polymorphism and hepatic transforming growth factor *β*1 expression in recurrent hepatitis C after liver transplantation. *Cytokine*.

[36] Promrat K, McDermott DH, Gonzalez CM (2003). Associations of chemokine system polymorphisms with clinical outcomes and treatment responses of chronic hepatitis C. *Gastroenterology*.

[37] Abbas Z, Moatter T (2003). Interleukin (IL) 1beta and IL-10 gene polymorphism in chronic hepatitis C patients with normal or elevated alanine aminotransferase levels. *The Journal of the Pakistan Medical Association*.

[38] Tanaka Y, Nishida N, Sugiyama M (2009). Genome-wide association of IL28B with response to pegylated interferon-*α* and ribavirin therapy for chronic hepatitis C. *Nature Genetics*.

[39] Li JH, Lao XQ, Tillmann HL (2010). Interferon-lambda genotype and low serum low-density lipoprotein cholesterol levels in patients with chronic hepatitis C infection. *Hepatology*.

[40] Abe H, Ochi H, Maekawa T (2010). A common variation of IL28 affects gamma-GTP levels and inflammation of the liver in chronically infected hepatitis C virus patients. *Journal of Hepatology*.

[41] Kurt-Jones EA, Popova L, Kwinn L (2000). Pattern recognition receptors TLR4 and CD14 mediate response to respiratory syncytial virus. *Nature Immunology*.

[42] Alexopoulou L, Holt AC, Medzhitov R, Flavell RA (2001). Recognition of double-stranded RNA and activation of NF-*κ*B by Toll-like receptor 3. *Nature*.

[43] Breiman A, Grandvaux N, Lin R (2005). Inhibition of RIG-I-dependent signaling to the interferon pathway during hepatitis C virus expression and restoration of signaling by IKK*ε*. *Journal of Virology*.

[44] Saito T, Owen DM, Jiang F, Marcotrigiano J, Gale M (2008). Innate immunity induced by composition-dependent RIG-I recognition of hepatitis C virus RNA. *Nature*.

[45] Otsuka M, Kato N, Moriyama M (2005). Interaction between the HCV NS3 protein and the host TBK1 protein leads to inhibition of cellular antiviral responses. *Hepatology*.

[46] Séne D, Levasseur F, Abel M (2010). Hepatitis C virus (HCV) evades NKG2D-dependent NK cell responses through NS5A-mediated imbalance of inflammatory cytokines. *PLoS Pathogens*.

[47] Crotta S, Brazzoli M, Piccioli D, Valiante NM, Wack A (2010). Hepatitis C virions subvert natural killer cell activation to generate a cytokine environment permissive for infection. *Journal of Hepatology*.

[48] Yoon JC, Shiina M, Ahlenstiel G, Rehermann B (2009). Natural killer cell function is intact after direct exposure to infectious hepatitis C virions. *Hepatology*.

[49] Golden-Mason L, Madrigal-Estebas L, McGrath E (2008). Altered natural killer cell subset distributions in resolved and persistent hepatitis C virus infection following single source exposure. *Gut*.

[50] Morishima C, Paschal DM, Wang CC (2006). Decreased NK cell frequency in chronic hepatitis C does not affect ex vivo cytolytic killing. *Hepatology*.

[51] Koziel MJ (2006). NK cells: natural born killers in the conflict between humans and HCV. *Hepatology*.

[52] Golden-Mason L, Rosen HR (2006). Natural killer cells: primary target for hepatitis C virus immune evasion strategies?. *Liver Transplantation*.

[53] Takehara T, Hayashi N (2005). Natural killer cells in hepatitis C virus infection: from innate immunity to adaptive immunity. *Clinical Gastroenterology and Hepatology*.

[54] Dolganiuc A, Oak S, Kodys K (2004). Hepatitis C core and nonstructural 3 proteins trigger toll-like receptor 2-mediated pathways and inflammatory activation. *Gastroenterology*.

[55] Riordan SM, Skinner NA, Kurtovic J (2006). Toll-like receptor expression in chronic hepatitis C: correlation with pro-inflammatory cytokine levels and liver injury. *Inflammation Research*.

[56] Abe T, Kaname Y, Hamamoto I (2007). Hepatitis C virus nonstructural protein 5A modulates the toll-like receptor-MyD88-dependent signaling pathway in macrophage cell lines. *Journal of Virology*.

[57] Koziel MJ, Wong DKH, Dudley D, Houghton M, Walker BD (1997). Hepatitis C virus—Specific cytolytic T lymphocyte and T helper cell responses in seronegative persons. *Journal of Infectious Diseases*.

[58] Koziel MJ (1997). The role of immune responses in the pathogenesis of hepatitis C virus infection. *Journal of Viral Hepatitis*.

[59] Rice CM, Walker CM (1995). Hepatitis C virus-specific T lymphocyte responses. *Current Opinion in Immunology*.

[60] Cramp ME, Rossol S, Chokshi S, Carucci P, Williams R, Naoumov NV (2000). Hepatitis C virus-specific T-cell reactivity during interferon and ribavirin treatment in chronic hepatitis C. *Gastroenterology*.

[61] Akyüz F, Polat N, Kaymakoglu S (2005). Intrahepatic and peripheral T-cell responses in genotype 1b hepatitis C virus-infected patients with persistently normal and elevated aminotransferase levels. *World Journal of Gastroenterology*.

[62] Hori S, Nomura T, Sakaguchi S (2003). Control of regulatory T cell development by the transcription factor Foxp3. *Science*.

[63] Marshall NA, Vickers MA, Barker RN (2003). Regulatory T cells secreting IL-10 dominate the immune response to EBV latent membrane protein 1. *Journal of Immunology*.

[64] Ulsenheimer A, Gerlach JT, Gruener NH (2003). Detection of functionally altered hepatitis C virus-specific CD4^+^ T cells in acute and chronic hepatitis C. *Hepatology*.

[65] Beilharz MW, Sammels LM, Paun A (2004). Timed ablation of regulatory CD4^+^ T cells can prevent murine AIDS progression. *Journal of Immunology*.

[66] Kondo Y, Ueno Y, Kobayashi K (2010). Hepatitis B virus replication could enhance regulatory T cell activity by producing soluble heat shock protein 60 from hepatocytes. *Journal of Infectious Diseases*.

[67] Kondo Y, Kobayashi K, Ueno Y (2006). Mechanism of T cell hyporesponsiveness to HBcAg is associated with regulatory T cells in chronic hepatitis B. *World Journal of Gastroenterology*.

[68] Bolacchi F, Sinistro A, Ciaprini C (2006). Increased hepatitis C virus (HCV)-specific CD4*+*CD25*+* regulatory T lymphocytes and reduced HCV-specific CD4*+* T cell response in HCV-infected patients with normal versus abnormal alanine aminotransferase levels. *Clinical and Experimental Immunology*.

[69] Marinho R, Pinto R, Santos ML, De Moura MC (2002). Evidence for prostaglandin-producing supressor cells in HCV patients with normal ALT. *Digestive Diseases and Sciences*.

[70] Germain RN (1994). MHC-dependent antigen processing and peptide presentation: providing ligands for T lymphocyte activation. *Cell*.

[71] Sadegh-Nasseri S, Stern LJ, Wiley DC, Germain RN (1994). MHC class II function preserved by low-affinity peptide interactions preceding stable binding. *Nature*.

[72] Doherty PC, Zinkernagel RM (1975). Enhanced immunological surveillance in mice heterozygous at the H-2 gene complex. *Nature*.

[73] Carrington M, Nelson GW, Martin MP (1999). HLA and HIV-1: heterozygote advantage and B*35-Cw*04 disadvantage. *Science*.

[74] Mangia A, Santoro R, Piattelli M (2004). IL-10 haplotypes as possible predictors of spontaneous clearance of HCV infection. *Cytokine*.

[75] Paladino N, Fainboim H, Theiler G (2006). Gender susceptibility to chronic hepatitis C virus infection associated with interleukin 10 promoter polymorphism. *Journal of Virology*.

[76] Abe H, Hayes CN, Ochi H (2011). IL28 variation affects expression of interferon stimulated genes and peg-interferon and ribavirin therapy. *Journal of Hepatology*.

